# Gender Differences in the Pathogenesis and Risk Factors of Hepatocellular Carcinoma

**DOI:** 10.3390/biology12070984

**Published:** 2023-07-11

**Authors:** Riccardo Nevola, Giovanni Tortorella, Valerio Rosato, Luca Rinaldi, Simona Imbriani, Pasquale Perillo, Davide Mastrocinque, Marco La Montagna, Antonio Russo, Giovanni Di Lorenzo, Maria Alfano, Maria Rocco, Carmen Ricozzi, Klodian Gjeloshi, Ferdinando Carlo Sasso, Raffaele Marfella, Aldo Marrone, Loreta Anesti Kondili, Nicolino Esposito, Ernesto Claar, Domenico Cozzolino

**Affiliations:** 1 Department of Advanced Medical and Surgical Sciences, University of Campania “Luigi Vanvitelli”, 80138 Naples, Italy; giotortorella95@gmail.com (G.T.); luca.rinaldi@unicampania.it (L.R.); simo.imbriani@gmail.com (S.I.); m.lamontagna92@libero.it (M.L.M.); giuann86@gmail.com (G.D.L.); maria.alfano2@libero.it (M.A.); rocco.maria92@gmail.com (M.R.); carmenricozzi28@gmail.com (C.R.); klodian87@yahoo.it (K.G.); ferdinandocarlo.sasso@unicampania.it (F.C.S.); raffaele.marfella@unicampania.it (R.M.); aldo.marrone@unicampania.it (A.M.); domenico.cozzolino@unicampania.it (D.C.); 2 Liver Unit, Ospedale Evangelico Betania, 80147 Naples, Italy; valeriorosato@gmail.com (V.R.); pasqualeperillo@hotmail.it (P.P.); davidemastrocinque4@gmail.com (D.M.); nicolinoe@gmail.com (N.E.); ernestoclaar@gmail.com (E.C.); 3 Department of Mental Health and Public Medicine, University of Campania “Luigi Vanvitelli”, 80138 Naples, Italy; antonio.russo2@unicampania.it; 4 Center for Global Health, Istituto Superiore di Sanità, 00161 Rome, Italy; loreta.kondili@iss.it

**Keywords:** hepatocellular carcinoma, HCC, sex hormones, androgens, estrogens, gender

## Abstract

**Simple Summary:**

Significant gender disparities have been highlighted in the incidence, aggressiveness, and prognosis of HCC. A different epidemiological distribution of the risk factors of liver damage and, above all, the actions of sex hormones are at the basis of these differences. Accurate knowledge of gender disparities in HCC would lead to adequate surveillance strategies and the potential implementation of current treatment schemes.

**Abstract:**

Several chronic liver diseases are characterized by a clear gender disparity. Among them, hepatocellular carcinoma (HCC) shows significantly higher incidence rates in men than in women. The different epidemiological distribution of risk factors for liver disease and HCC only partially accounts for these gender differences. In fact, the liver is an organ with recognized sexual dysmorphism and is extremely sensitive to the action of androgens and estrogens. Sex hormones act by modulating the risk of developing HCC and influencing its aggressiveness, response to treatments, and prognosis. Furthermore, androgens and estrogens are able to modulate the action of other factors and cofactors of liver damage (e.g., chronic HBV infection, obesity), significantly influencing their carcinogenic power. The purpose of this review is to examine the factors related to the different gender distribution in the incidence of HCC as well as the pathophysiological mechanisms involved, with particular reference to the central role played by sex hormones.

## 1. Introduction

Hepatocellular carcinoma (HCC) is the sixth most common neoplasm in the world, the third in order of mortality, with 906,000 cases and 830,000 deaths per year [1]. Chronic inflammatory processes and liver cirrhosis represent the most fertile substrates for the genesis of HCC [2]. Among the main risk factors, hepatitis B (HBV) and C (HCV) viruses, aflatoxin contaminating some cereals, alcohol abuse, obesity, and diabetes mellitus are, today, most frequently associated with the development of liver neoplasms. The different prevalences of risk factors account for a heterogeneous global distribution. Regions with a high prevalence of major hepatotropic viruses (East and South-East Asia, West and North Africa) show high prevalence rates of HCC, while relatively lower rates are found in Northern Europe, America, and Australia, where the main risk factors, on the other hand, are linked to the high prevalence of alcoholism and, above all, of metabolic syndrome secondary to the typical lifestyle of industrialized countries (high-calorie diet, low physical activity) [3]. However, a substantial global change in the distribution and relative impact of risk factors for liver cirrhosis and HCC is currently underway. In fact, thanks to the diffusion of vaccines for HBV and the progressive improvement of therapies for both chronic HCV and HBV infections, the viral etiology is becoming less and less prevalent, in favor of a progressive increase in the forms linked to metabolic associated fatty liver disease (MAFLD) [1,4]. The different prevalence of risk factors also accounts for an inhomogeneous distribution between the two genders. In fact, the male gender shows incidence rates of HCC at least 2 or 3 times higher than female ones [1,5]. Although the imbalance of risk factors (e.g., alcoholism) and cofactors (e.g., cigarette smoking) in favor of males weighs on the different gender distribution, other elements seem to be crucial. In this regard, sex hormones play a significant role. In fact, the liver is now recognized as a sexually dysmorphic organ that is extremely susceptible to interactions with estrogens and androgens [6]. Therefore, both exogenous (lifestyle) and endogenous (sexual hormones) factors contribute to the higher incidence of HCC in men. Furthermore, clinical course and prognosis of HCC in women appear significantly better than those in men.

The purpose of this review is to examine the factors related to the different gender distribution in the incidence and prognosis of HCC, as well as pathophysiological mechanisms involved, and the clinical implications that this heterogeneity determines.

## 2. Epidemiological and Clinical Gender Difference in HCC

HCC accounts for over 80% of primary liver tumors. Its incidence is 2 to 3 times higher in men than in women, depending on region and clinical trial (Table 1). This difference is greatest in some European countries (such as France and Malta, with a male/female ratio of 5.0 and 4.8, respectively) and minimal, up to an equal ratio between the two sexes, in other countries (Uganda, Costa Rica, Ecuador, and Columbia) [1]. In 2020, liver cancer accounted for 6.3% (632,320 cases) of newly diagnosed cancers in men and 3.0% (273,357 cases) in women. HCC in the male gender usually occurs at an earlier age than in women [7]. In particular, the development of HCC most likely occurs at ages of 50–69 in men, whereas the incidence appears similar between ages 50–69 and ≥ 70 years in women. Although the trend is dynamic, to date the forms of HCC secondary to alcohol and chronic hepatitis B virus (HBV) infection are more prevalent in men, whereas HCV-related and metabolic-related HCC are more prevalent in women (Table 1) [7].

In addition to significant epidemiological differences, there are relevant clinical and prognostic differences between the two sexes. Significant gender differences in course and prognosis have already been demonstrated in other types of neoplasms (for example: melanoma, lung carcinomas, gastrointestinal carcinomas) [8,9,10]. As concerns HCC, in women it tends to have a less aggressive course than in men, resulting in a better prognosis and a mortality rate that is 2–3 times lower (Table 1) [1,11,12,13,14]. In particular, women have a better overall survival (OS) [13,14] and a lower recurrence rate, with median disease-free survival (DFS) of 19.5 months compared with 4.5 months for men [14]. In a recent retrospective analysis of 1110 HCC cases (of which 23.5% were female), Rich et al. [12] showed that the tumor developed at a younger age in men than in women (59.2 vs. 62.5 years, *p* < 0.001) and the latter had higher proportion of early-stage tumors at diagnosis. In multivariable analysis, female sex was independently associated with lower mortality rate, early tumor detection, and response to first HCC treatment.

The lesser aggressiveness of liver tumors in women seems to be related to the reduced size of the tumors [13,14]. The HCC encapsulation rate (predictor of lower recurrence [15]) is also significantly higher in women than in men [14]. At presentation, HCC in women shows less tendency to multifocality, macrovascular invasion, and metastasis than in men and is more frequently diagnosed in the phase of compensated liver disease [11,12,13,14,16,17]. It follows that diagnosis at an earlier stage of the disease makes women more susceptible to treatments with curative purposes (liver transplantation, surgical resection and ablation) [18]. Furthermore, histological review of HCCs subjected to resection has shown that in women the frequency of positive resection margins for neoplasia is significantly lower [14]. Overall, the diagnosis of an early-stage HCC contributes to better prognosis and lower recurrence rates in women than in men [15]. In this regard, Liang et al. [19] recently highlighted that male patients showed significantly higher risk for HCC recurrence than females (hazard ratio, HR: 1.480; 95% confidence interval, IC: 1.084–2.020). In particular, male gender proved to be an independent risk factor for early (but not late) recurrence (odds ratio, OR: 1.864; 95% CI: 1.215–2.936). 

Although men show, on average, less compliance than women with surveillance programs [20], contributing to a late diagnosis, and higher risk factors for HCC (see next paragraph), other variables may contribute to determining the lower aggression and better prognosis of HCC in females. In this regard, it is important to underline how the differences between the two genders in the course and prognosis of HCC are more evident in younger age groups, whereas in older patients these differences tend to decrease [12,21,22]. In fact, although women < 65 years demonstrate a better OS than men (18.3 vs. 11.2 months, *p* = 0.02), the two sexes show similar rates of OS over 65 years (15.5 vs. 15.7 months, *p* = 0.45) [12]. It therefore appears probable that this trend is due to the greater impact of female hormones in the premenopausal period. The role of sex hormones on the prognosis of HCC is confirmed by the impact of estrogen intake. Indeed, oral contraceptive use has been shown to be an independent positive prognostic factor in patients with HCC [23]. In a case-control study, Hassan et al. [24] showed that use of estrogens is associated with an estimated adjusted odds ratio for HCC of 0.53 (95% CI: 0.32–0.88) and a significant reduction in the risk of death from HCC (hazard ratio, HR: 0.55; 95% CI: 0.40–0.77) with a median OS of 33.5 months for estrogen users and 24.1 months for non-users. However, although numerous studies performed in South-East Asia agree on the better course and higher survival rates of women with HCC compared to men [13,18,23,25], some Western retrospective studies do not confirm this data [16,26]. It therefore appears conceivable how racial/ethnic disparities can simultaneously influence HCC prognosis [27].

## 3. Gender Differences in Risk Factors of Hepatocellular Carcinoma

Risk factors for chronic liver damage show significant differences between the two genders. Historically, men are more prone to habits such as alcohol, cigarette smoking, high-calorie diets, and drug use than women. However, in recent decades there has been a significant increase in such behaviors in the female gender, with an increase in cases of chronic hepatitis, cirrhosis, and HCC among women. Although these gender disparities are progressively less pronounced, they still account, at least in part, for the different risk of developing HCC between the two genders (Table 1). 

### 3.1. Alcohol

Alcohol abuse remains one of the major non-viral risk factors for chronic liver injury and development of HCC [3,28]. In addition to the risk induced by hepatic fibrosis and cirrhosis, it also favors hepatocarcinogenesis by producing reactive oxygen species (ROS) with secondary oxidative stress and potential alterations of hepatocyte DNA [28,29]. In patients with alcohol-related cirrhosis, the annual incidence of HCC ranged from 0.9% to 5.6% [30]. Overall, alcohol appears to be responsible for 26% of HCC cases worldwide [3], with a neoplastic risk directly proportional to the amount of alcohol ingested [31]. Historically, men are greater alcohol consumers than women, which partly explains the increased risk of HCC demonstrated in men (Table 1) [3]. However, the gap between the two sexes is less and less pronounced due to a significant increase in alcohol consumption among women, particularly in industrialized countries [30]. Furthermore, due to the different pharmacokinetics of ethanol, women appear more susceptible to alcohol-induced liver injury than men [32]. Despite this net of greater alcohol consumption in men, among patients with alcohol-related cirrhosis, the incidence of HCC is lower in women [30]. In a cohort of 8482 patients with alcohol-induced liver cirrhosis, Jepsen et al. [33] showed that the HCC incidence was 5.8 and 0.7 (per 1000 patient/year) in men and women, respectively. Recently, Ganne-Carrié et al. [34] finally confirmed that among patients with alcohol-induced liver cirrhosis, male gender is significantly associated with the risk of HCC occurrence. Therefore, given the greater consumption of alcohol, other factors contribute to the higher incidence of HCC among men.

### 3.2. Obesity and Metabolic Syndrome

More than COVID-19, the real pandemic of the third millennium is that of obesity, type 2 diabetes mellitus (T2DM), and metabolic syndrome [35]. In Western countries, and by now also in less industrialized countries, the spread of unbalanced lifestyles (high-calorie diets, low physical activity) is causing a significant increase in the prevalence of metabolic syndrome. MAFLD is one of the most frequent organ manifestations of metabolic syndrome, with an overall prevalence of 32.4% [36,37]. Since the significant reduction in viral etiologies, it represents one of the most frequent causes of liver cirrhosis and HCC to date [4,38]. Furthermore, in patients with liver cirrhosis of other etiologies, the presence of obesity and/or diabetes mellitus increases the oncogenic risk by 2 to 4 times [39,40]. It is estimated that for every 5 kg/m2 increase in body mass index (BMI) there is a 30% increase in the risk of developing liver cancer (relative risk, RR: 1.30; 95% CI: 1.16–1.46) [41]. In particular, about 9% of the overall cases of HCC can be attributed to obesity and 7% to the presence of T2DM [3]. Hormonal alterations induced by insulin resistance and chronic systemic inflammation probably represent the pathogenetic mechanisms responsible for the relationship between obesity and cancer [42].

As expected due to a biologically driven higher amount of body fat, the worldwide prevalence of obesity is higher in women than in men, regardless of age (Table 1) [43]. It increased significantly between 1980 and 2019 in both genders (from 6% to 15.7% in women and from 3.2% to 12.2% in men). Although in men the prevalence of obesity increases for all income levels, in women obesity is positively related to income in low-income countries and inversely related in high-income ones. In these countries, the gender gap tends to narrow since the increase in male obesity is faster than female obesity. Unlike obesity, significant male predominance in the prevalence of T2DM is instead reported [44,45,46]. Although women are more frequently obese, an increase in visceral (rather than subcutaneous) adipose tissue prevails more often in men due to the high tissue expression of androgen receptors and the low expression of estrogen receptors [47]. Visceral obesity, more frequent in men, closely correlates with the development of insulin resistance and accounts for male predominance in the prevalence of T2DM [46].

A careful analysis performed by Sung et al. [41] for 2012 estimated that the number of cases of liver cancer attributable to excess weight was 84,800, that is, 11% more than expected. However, although obesity overall tends to prevail in females, the total number of HCC related to excess body weight was 30,200 cases/year in women and 54,600 cases/year in men. Furthermore, the impact of obesity on HCC outcomes seems to take the opposite direction compared to the prevalence data. Indeed, according to the meta-analysis of Gupta et al. [39], magnitude of increased HCC-related mortality was higher in obese men (adjusted hazard ratio—aHR 2.50; 95% CI: 2.02–3.09) than obese women (aHR 1.45; 95% CI: 1.08–1.97).

### 3.3. Viral Hepatitis

Viral hepatitis resulting from chronic infection with HCV, HBV, and hepatitis D virus (HDV) are among the main determinants of liver cirrhosis and HCC [48]. If all major hepatotropic virus infections are able to induce HCC through mechanisms of liver damage and regeneration leading to cirrhosis, HBV shows a further intrinsic oncogenic potential linked to the peculiar interactions between virus and host [49]. Globally, the proportion of HCC cases attributable to HBV and HCV infection is 44% and 21%, respectively [3].

Although historically there has been a predominance of the male gender in the prevalence of viral hepatitis, the secondary intrafamilial diffusion and the increase in the number of people who use drugs (PWUD) among women has in fact reduced the disparity in gender distribution [48]. Despite this, there are some differences in the distribution of HCC cases secondary to viral hepatitis, with wide variations in relation to the geographical area involved [50]. Overall, HBV-related HCC appears more prevalent in men, whereas HCV-related HCC appears more prevalent in women to date (Table 1) [7]. However, thanks to the availability of effective therapies (HBV, HCV) and vaccines (HBV), the proportion of HCC secondary to viral hepatitis is progressively decreasing in favor of metabolic forms [1,51,52]. Therefore, viral hepatitis will tend to affect less and less gender differences in the epidemiology of liver cancer.

### 3.4. Other Causes of Cirrhosis

Primary biliary cholangitis and autoimmune hepatitis are rare causes of chronic liver injury potentially progressing to liver cirrhosis and HCC. These etiologies are clearly prevalent in the female sex, with a female/male ratio up to 9/1 (Table 1) [53]. However, the proportion of HCC attributable to these etiologies is low (<5% worldwide), with minimal impact on global gender differences.

### 3.5. Cigarette Smoking

Smoking is associated with de novo malignancies, including lung, oropharyngeal, esophageal, and laryngeal cancer, among others. However, cigarette smoking also contributes to carcinogenesis in HCC [54]. It is estimated that smoking contributes to 13% of all HCC cases worldwide [3]. Compared with never smokers, the RR for HCC is 1.51 (95% CI: 1.37–1.67) for current smokers and 1.12 (95% CI: 0.78–1.60) for previous smokers [55]. Furthermore, a significant relationship between dose (pack/year) and increased risk of liver cancer appears likely [56]. The carcinogens contained in tobacco can induce injury-mediated changes in gene expression in genetically predisposed individuals [54]. In particular, tobacco-specific nitrosamines are the cause of DNA adducts, expressions of growth factors, and chronic systemic inflammation, underlying the mechanisms of cell proliferation and metastases [57]. 

The cigarette smoking habit is predominant in the male gender in all regions of the world, although this disparity is greatest in Asia and Africa (Table 1) [3]. Globally, the prevalence of current smokers is 32.7% among men and 6.62% among women, with wide variations in relation to the geographical area [58]. It has an impact on the development of HCC that is more significant for men than for women.

## 4. Genetic Gender Differences in the Risk of HCC Development

Beyond the different gender distribution of modifiable risk factors, a series of non-modifiable risk factors for HCC linked to individual genetic predispositions and differences between male and female sexes have been hypothesized. However, the knowledge of the different genetic predispositions between the two sexes in the development of HCC is still very limited.

Although few protein-coding genes are present in the male-specific region of the Y chromosome, Y chromosome genes may play a role in the different incidence and aggressiveness of HCC between the sexes. The sex-determining region Y (SRY) is a gene located on the Y chromosome that encodes for the protein testis-determining factor (TDF), an important transcription factor implicated in male sexual differentiation [59]. In addition to its role in the development of male sexual characteristics, TDF is also involved in the differentiation of other tissues, including those of the peripheral and central nervous system, gastrointestinal tract, and liver [60]. Some evidence also suggests a role of SRY in the development of HCC. Xue et al. [61] evaluated SRY expression in HCC cell lines, highlighting an elevated expression in the neoplastic tissue. Furthermore, high levels of tissue expression of SRY would correlate with worse prognosis. Liu et al. [62], through in vivo preclinical studies performed on transgenic mice characterized by overexpression of SRY and treated with N-diethylnitrosamine (DEN, a promoter of HCC development), indeed showed that overexpression of SRY in male mice promoted hepatocarcinogenesis in 84% of male mice, activating the Sox9 and PDGFRα/PI3K/Akt pathway. Sox9 is a gene implicated in testis development, but also transiently expressed by hepatoblasts and biliary endothelial cells at the early stage of embryonic liver development and by regenerating hepatocytes after liver injury [63]. Overexpression of SRY could induce the activation of Sox9, making hepatocytes acquire potentialities similar to hepatic progenitor cells and induce inflammation, fibrosis, and cell proliferation processes up to hepatocarcinogenesis. In addition to higher incidence of HCC, the data obtained by Liu et al. [62] indicate that male mice overexpressing SRY develop larger tumors and more inflammation and tissue fibrosis than controls. Furthermore, female mice with aberrant expression of SRY (due to probable chromosomal translocation) also show higher probability of tumor development than the wild type. Overall, these data confirm the role of the male-specific gene SRY in the development of HCC in men and could explain in part the gender differences in the incidence and behavior of liver tumors. However, since female transgenic mice showed lower HCC occurrence than male transgenic mice (55.6% vs. 100%), it seems likely that there is an influence of other gender-related factors (e.g., estrogen) on the promotion of hepatocarcinogenesis induced through SRY activation.

Although unable to encode proteins, long noncoding RNAs (LncRNAs) are RNA transcripts able to significantly influence gene expression and regulate processes of cell differentiation, proliferation, and migration [64]. LncRNA FTX is transcribed by the FTX gene (five prime to Xist) located at the level of the X-chromosome [65]. Through the modulation of the Xist gene, LncRNA FTX is able to modulate the inactivation of the X chromosome. Due to its role, LncRNA FTX is involved in the development and progression of several types of cancer [66]. In the HCC setting, LncRNA FTX acts as a tumor suppressor, inhibiting HCC cell growth and metastasis [67]. In fact, a reduced expression of LncRNA FTX correlates with the development of cancer and is associated with worse prognosis, whereas higher LncRNA FTX expression correlates with a longer survival. Indeed, in NAFLD (nonalcoholic fatty liver disease) mouse models, the downregulation of LncRNA FTX favored the development of HCC, while its upregulation promoted M1 polarization of liver Kupffer cells, preventing liver damage and inhibiting the malignant transformation of hepatocytes [68]. LncRNA FTX acts as a negative regulator of the Wnt/β-catenin signaling by inhibiting HCC cell epithelial-mesenchymal transition and repressing tumor invasion and metastasis [67]. In hepatocytes, LncRNA FTX shows a significantly higher expression in women than in men. It follows that in females the high expression of LncRNA FTX appears to be a protective factor against hepatocarcinogenesis, reducing the risk of developing HCC and, when it happens, favoring less aggressiveness (local and systemic).

Beyond the direct role played by some genes in determining a greater male predisposition to the development of HCC, the level of gene expression can be significantly influenced by other factors (for example sex hormones), modulating the oncogenic risk. Such pathways will be discussed below.

## 5. Impact of Sex Hormones on the Development of Hepatocellular Carcinoma

In the liver, the presence of estrogen and androgen receptors has been demonstrated for several decades [69]. These hormones play a key role in many hepatic physiological functions, taking part in lipid and glucose metabolism regulation and having a protective role on inflammation and fibrosis, making the liver an organ with recognized sexual dimorphism [70]. Due to this main role in the regulation of hepatic physiological processes, sex hormones are also involved in the development of pathologies, in particular HCC.

### 5.1. Estrogens

Estrogens are steroid hormones mainly produced in the ovary, placenta, corpora lutea, adrenal glands, and adipose tissue and are responsible for the development of the female reproductive system and secondary sexual characteristics [71,72]. Four types are recognized: estrone (E1), 17β-estradiol (E2), estriol (E3), and estetrol (E4). These hormones exert their action through binding with estrogen receptors (ER) α and β, located in the nucleus and cell membrane, and with the membrane receptor G-protein coupled estrogen receptor (GPER) [73,74]. Therefore, estrogens are able to activate the intracellular signaling cascade both through binding with ERα, Erβ, and GPER receptors, and through entry into the plasma membrane, interacting directly with intracellular ERα and ERβ. The receptor activation induces transcriptional processes and/or signaling events able to modulate the gene expression through mechanisms that predict (genomic) or do not predict (nongenomic) the direct link between the receptor complex and specific DNA sequences [73]. Genomic effects are induced by migration of the estrogen receptor complexes towards the nucleus and direct interaction with specific DNA sequences known as estrogen response elements (EREs) [75]. In addition, approximately one-third of genes whose transcription is estrogen-dependent have been shown to lack specific ERE regions [76]. In fact, these hormones have the ability to transduce the signal even in the absence of a direct link with the target DNA. In fact, they can interact with specific transcription factors, such as activator protein-1 (AP-1) and stimulating protein-1 (Sp-1), able to act on a multitude of target genes and significantly amplify the ability of estrogen-mediated gene regulation [73]. Nongenomic effects are based on indirect regulation of gene expression through complex intracellular signaling events, mainly involving phospholipase C (PLC)/protein kinase C (PKCs), phosphatidyl inositol 3 kinase (PI3K)/Akt kinase, Ras /Raf/MAPK, and cAMP/protein kinase A (PKA). Finally, numerous interactions between genomic and nongenomic signal transduction pathways have been demonstrated [73,77].

Estrogens play a role in numerous functions, including the development of primary and secondary female sexual characteristics, the regulation of reproductive mechanisms and the menstrual cycle, bone metabolism, cholesterol mobilization, and inflammation control, also influencing the function of other systems (e.g., cardiovascular and nervous) [73,78,79]. In males, a low level of estrogen is essential for sperm maturation and erectile function. Due to the role played in the modulation of numerous functions, their dysregulation is crucial in the pathogenesis of a variety of diseases, including cardiovascular (e.g., atherosclerosis, arterial hypertension), metabolic (e.g., metabolic syndrome, dyslipidemia), bone (e.g., postmenopausal osteoporosis), and central nervous system (e.g., Alzheimer’s disease, psychiatric disorders) diseases [80,81,82,83,84,85]. In oncology, estrogens play a key role in the development of breast cancer (where the binding of female hormones with ERα stimulates cell proliferation, while the binding with ERβ plays an antiproliferative role), ovarian cancer, and endometrial cancer, but also in the incidence of mesothelioma, meningioma, prostate cancer, renal cell carcinoma, colorectal, and lung cancer [86].

**Figure 1:**
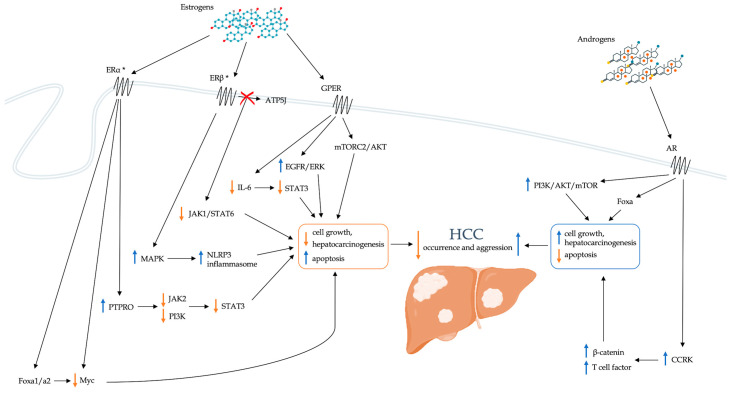
Schematic representation of the direct role of sex hormones in modulating the risk of developing HCC. AKT: Ak strain transforming; AR: androgen receptor; ATP5J: ATPase-coupling factor 6; CCRK: cell cycle-related kinase; EGFR: epidermal growth factor receptor; ER: estrogen receptor; ERK: extracellular signal-regulated kinase; Fox: forkhead box; GPER: G-protein coupled estrogen receptor; HCC: hepatocellular carcinoma; IL-6: interleukin-6; JAK: Janus kinase; MAPK: Mitogen-activated protein kinase; mTOR: mammalian target of rapamycin; mTORC2: mTOR complex 2; NLRP3: nucleotide-binding domain, leucine-rich–containing family, pyrin domain–containing-3; PI3K: phosphatidylinositol-3 kinase; PTPRO: receptor-type tyrosine-protein phosphatase O; STAT: signal transducer and activator of transcription. * ERs are present both at the level of the cell membrane and of the nucleus.

Finally, growing evidence is available about the impact of estrogens on the development of HCC. Both healthy livers and HCC livers express estrogen receptors. In animal models, the administration of estrogens can modulate cell proliferation and the risk of HCC development [87]. The influence of estrogen on the risk of developing HCC occurs at multiple levels. The related mechanisms are summarized in Figure 1.

#### 5.1.1. PTPRO

The receptor-type tyrosine-protein phosphatase O (PTPRO) is a member of the protein tyrosine phosphatases (PTPs) family that acts as a mediator of cell signaling pathways by inhibiting tumor proliferation and degeneration [88]. The PTPRO gene shows three ERE regions at the promoter level, strictly dependent on estrogens action and their binding to ERα [89]. Recent studies highlight its role as a tumor suppressor in different types of neoplasms, such as lung [90], renal [91], and colorectal cancer [92]. Indeed, PTPRO appears able to inhibit Janus kinase 2 (JAK2) and phosphoinositide 3-kinase (PI3K) dephosphorylation which are crucial in the activation of the transcription factor STAT3. Therefore, the suppressive role of PTPRO in cancer is due to STAT3 inactivation, which instead appears upregulated when PTPRO levels are reduced [93]. In the HCC setting, STAT3 plays a central role in the processes of development, progression, and metastasis [94]. Hou et al. [89] demonstrated that PTPRO levels are strongly reduced in HCC cell lines when compared with those in adjacent healthy tissues, resulting in STAT3 overexpression, and that tumor number and size were increased in PTPRO knockout mice. Furthermore, PTPRO levels in male adjacent tissue were lower than in female tissue [89]. Through ERα activation and binding to ERE regions, estrogens could induce PTPRO overexpression, favoring STAT3 inhibition. They would therefore act by reducing the risk of developing HCC and, when it develops, favoring less aggressiveness and consequently a better prognosis. The role of PTPRO would therefore contribute to the gender differences observed in the HCC setting.

#### 5.1.2. Foxa1 and Foxa2

Forkhead box (Fox) transcription factors are a family of transcription factors derived from the Fox genes and involved in hormonal and immune system regulation, embryogenesis, cell proliferation, and growth through the regulation of the epithelial-mesenchymal transition [95]. In oncology, they have a role in tumor development and in progression and metastasis processes, including breast, ovarian, and prostate cancer, and HCC [96]. Fox transcription factors are categorized into subclasses A to S [97]. Recently it has been highlighted that FoxC1 is able to promote the development of HCC and metastasis [98]. However, to date it has no known impact on gender differences in this setting. Conversely, Foxa1 and Foxa2, as well as being crucial in liver development and differentiation, are responsible for the sexual dysmorphism of HCC [99,100]. Li et al. [100] evaluated the role of these transcription factors in Foxa1- and Foxa2-deficient mice after DEN-induced hepatocarcinogenesis. The authors showed that in the absence of Foxa1/a2, the sexually dimorphic HCC is completely inverted and that Foxa1/a2-deficient females show greater size and more frequent multifocality than non-Foxa1/2-deficient controls. In deficient mice, coregulation of target genes by Foxa1/a2 and either the ERα or the androgen receptor was lost. Foxa and ERα modulate several pathways in resistance to HCC. In particular, the Myc oncogene seems crucial in the oncoprotection mechanisms exerted by Foxa and ERα. Myc is, in fact, significantly inhibited by Foxa and Erα, and is overexpressed in conditions with Foxa1/a2 deficiency. When Myc expression is suppressed, hepatocyte proliferation and the likelihood of neoplastic transformation are greatly reduced. In order to perform this, it has been hypothesized that a coregulation of both Foxa1/a2 and ERα is necessary. By itself, ERα does not appear to be able to inhibit Myc. Indeed, in absence of Foxa1/a2, estrogens would seem to favor hepatocarcinogenesis. Foxa1/2 gene polymorphisms are associated with decreased binding of Foxa2 and ERα to their targets in the liver and correlate with HCC development in women. In males, on the other hand, an opposite mechanism seems to take place. In Foxa1/a2-deficient male mice, there is a reduced incidence of HCC and lower tumor burden. It has been hypothesized that Foxa1/a2 and AR cooperate in the regulation of gene expression and that Foxa1/a2 are essential for androgen signaling in promoting HCC development in male mice. Overall, these data confirm that the estrogen-dependent resistance to the development of HCC in females and the androgen-dependent favorability in males are mediated by Foxa1/2. As already described for PTPRO, the role of Fox transcription factors also appears to be decisive for the sexual dimorphism of HCC.

#### 5.1.3. GPER

As already mentioned, GPER is a more recently discovered membrane estrogen receptor [74]. In addition to its already known functions, some studies have hypothesized a protective role of GPER against the development of HCC. GPER knockout mouse models indeed show a significant increase in inflammation (expressed by increased levels of IL-6) and liver fibrosis and accelerated hepatocarcinogenesis [101]. Furthermore, GPER levels are significantly lower in HCC compared with nontumor tissues. In HCC patients, GPER-positive patients more frequently show small tumor size, low serum alpha fetoprotein levels, and longer OS than GPER-negative patients [102]. The protective action of GPER against the development of HCC would depend on the ability to suppress the inflammatory response in the tumor microenvironment [101]. Furthermore, GPER is also able to modulate the estradiol-dependent expression of Sin1, the regulatory subunit of mTOR complex 2 (mTORC2) activity [103]. Via phosphorylation mechanisms, mTORC2 controls AKT activation through which GPER influences cell proliferation and hepatocarcinogenesis. Treatment with GPER-specific agonists has been shown to activate EGFR/ERK signaling pathways, thereby promoting apoptosis and inhibiting cell growth [102].

#### 5.1.4. Inflammation

The development of HCC is closely related to tissue inflammation [104]. In fact, the liver has a marked innate immunity characterized above all by a significant proportion of natural killer cells (NK) and macrophages (KCs, Kupffer cells) [105]. If the innate immune system provides a rapid initial response to a broad range of hepatic insults, the adaptive immune system provides a specific immune response against pathogenic noxae to which the liver has been previously exposed. However, chronic inflammatory stimuli (e.g., chronic HBV or HCV infection, alcohol, NAFLD) can activate hepatic stellate cells (HSC), which, by differentiating into myofibroblasts, determine collagen deposition and consequent tissue fibrosis [106]. Chronic liver damage and inflammation are the cause of cell regeneration and production of reactive oxygen species (ROS), with potential damage to hepatocyte DNA and development of procarcinogenic mutations, which ultimately can lead to HCC. If chronic inflammatory processes are crucial in hepatocarcinogenesis, the influence of sex hormones on the mechanisms of onset and persistence of inflammation could play a decisive role in developing HCC. The main pathways by which sex hormones influence the inflammatory response and the risk of HCC known to date are described below.

##### IL-6

Interleukin 6 (IL-6) is a multifunctional inflammatory cytokine involved in the development of inflammation and cell proliferation [107]. In the liver, it is produced by Kuppfer cells and is a significant inducer of acute phase and infection defense responses [108]. Furthermore, IL-6 acts as a hepatocyte mitogen, with a role in the mechanisms of liver regeneration and the development of neoplasms. IL-6 binding to the IL-6 receptor (IL-6R) activates the Janus kinase (JAK), stimulating phosphorylation and activating signal transducers and activators of transcription 3 (STAT3) [109]. Activation of the IL-6/STAT3 axis is responsible for the role of IL-6 in the processes of anti-apoptosis, proliferation, invasion, angiogenesis, and metastasis. In patients with HCC, IL-6 levels are significantly increased and correlate with the occurrence of HCC and prognosis [110]. In females, high serum IL-6 levels have been shown to be an independent risk factor for HCC development (hazard ratio, HR: 1.61) [111]. Naugler et al. [112] investigated the role of IL-6 in HCC gender differences. The authors noted that after administration of the carcinogen DEN serum, IL-6 levels increased more in males than in females. Furthermore, the inhibition of IL-6 is able to cancel the gender differences in hepatocarcinogenesis. After DEN administration, IL6 −/− knockout mice show less apoptosis, liver cell proliferation, and necrosis than wild type mice. Collectively, these data indicate that the development of HCC is associated with elevated IL-6 levels and that IL-6 contributes to gender differences. This hypothesis is confirmed by the evidence that some IL-6 polymorphisms have been associated with the onset of HCC [113]. The protective effect of the female gender would occur mainly among carriers of phenotypes characterized by high IL-6 production. Furthermore, since oophorectomy is able to cancel gender disparities, it is conceivable that estrogens play a crucial role in this setting [112]. Indeed, female sex hormones would be able to inhibit the production of IL-6 from KCs through the suppression of the transcription factors NF-kappaB and C/EBP-β [114], with a protective effect on the development of HCC in females.

##### Tumor-Associated Macrophages

Within the tumor microenvironment, tumor-associated macrophages (TAMs) have an active role in the genesis and progression of the tumor [115]. Macrophage activation consists of two pathways, the classic one (M1) and an alternative one (M2) [116]. Macrophages acquiring an M2 phenotype are able to infiltrate tumor tissues guided by tumor- and T-cell-produced cytokines and promote tumor growth. Yang et al. [117] showed that estrogens act by inhibiting the alternative activation pathway of TAMs. In particular, 17β-estradiol is able to prevent the binding between ERβ and ATPase-coupling factor 6 (ATP5J) through the suppression of IL4-mediated phosphorylation of the transcription factors JAK1 and STAT6, thus inhibiting the JAK1-STAT6 signaling pathway. In this way, 17β-estradiol could suppress tumor growth by regulating the macrophage’s polarization.

##### NLRP3

The NLRP3 inflammasome is an intracellular multiprotein complex mediating innate immunity that assembles in response to cellular insults [118]. When assembled, NLP3 activates caspase-1, which is responsible for the release of inflammatory cytokines (interleukin-1β–IL-1β, and inteleukin-18–IL-18) and the promotion of pyroptosis, that is, of inflammatory cell death resulting from the formation of pores on the cell membrane [119]. Wei et al. [120] analyzed the role of the NLRP3 inflammasome in the development and progression of HCC, and they found that the expression of the NLRP3 inflammasome was completely lost or significantly downregulated in HCC tissue. Due to the loss of the protective and antiproliferative functions of the inflammasome, this deregulation of the NLRP3 in HCC correlates with poor histological differentiation and with increased tumor progression. The same authors also demonstrated that treatment with 17β-estradiol can lead to significant upregulation of the NLRP3 inflammasome via the E2/ERβ/MAPK pathway [121]. Therefore, estrogens would be able to suppress the development and progression of HCC also through stimulation of the NLRP3 inflammasome.

### 5.2. Androgens

Androgens are steroidal sex hormones essential for both sexes, however the serum concentration is significantly higher in men than in women [122]. These hormones are produced by adult female ovaries, male heads, and in adrenal glands, playing a central role in the development of sexual characteristics and mechanisms of reproduction. Furthermore, androgens are also necessary precursors for estrogen biosynthesis. In adult men, testosterone is the predominantly represented androgen hormone, which can be converted to dihydrotestosterone (DHT), the most potent endogenous androgen with 5 to 10 times higher affinity for the androgen receptor (AR) than that of testosterone [123]. The binding of androgens to their AR causes a change in receptor conformation with translocation of the complex into the nucleus and interaction with specific DNA sequences, the androgen response elements (AREs) [124]. Ultimately these regulate the transcription of a series of genes, including those responsible for cell growth and survival, which therefore appear sensitive to androgenic action.

Although they have a less defined role than estrogens, androgens also play important functions in the pathogenesis of HCC (Figure 1). Indeed, AR expression is increased in HCC tissue compared with normal liver, and mice lacking hepatic ARs develop HCC later and less frequently than wild-type mice [125]. Furthermore, AR overexpression is associated with disease progression and is an independent predictor of OS [126]. In particular, AR overexpression alters 67% of the AR target genes in HCC cells and promotes cell growth and oncogenic proliferation. Differently from prostate cancer, mechanistic target of rapamycin (mTOR) protein, a key member of the PI3K-AKT-mTOR signaling pathway frequently hyperactivated in several malignancies, stimulates AR transcriptional activity in HCC. The frequent activation of mTOR signaling pathways could represent a plausible molecular mechanism for nuclear AR overexpression in HCC. Furthermore, according to Feng et al. [127], AR activation would lead to greater transcription of the cell cycle-related kinase (CCRK) regulator, a critical mediator of AR signaling that appears markedly increased in HCC. CCRK would drive the processes of hepatocarcinogenesis by stimulating the signaling cascade mediated by β-catenin and T-cell factor. Overexpression of CCRK seems to be able to determine AR-induced cell cycle stimulation, hepatocellular proliferation, and malignant transformation.

In addition to the role directly played by male sex hormones and RA in the pathogenesis of HCC and its male predominance, androgens are also able to significantly enhance the oncogenic power of HBV infection [128]. The role of the androgen/AR axis in the pathogenesis of HCC during chronic HBV infection will be discussed in the next section.

In light of the male predominance in HCC incidence and the role of androgens and AR in oncogenic proliferation, anti-androgen and anti-AR therapies have been tested in the treatment of liver cancer. However, the results obtained were unsatisfactory [129]. Despite the relevant role played by the androgen/AR axis in the pathogenesis of HCC, probably only a small proportion of liver cancers (about one third) overexpress AR and could be responsive to AR inhibition [126]. In order to maximize treatments for HCC, the tumor biology in each patient (assessed by liver biopsy or, hopefully, by liquid biopsy) should guide the personalization of treatment, tending increasingly towards precision medicine [130]. 

### 5.3. Influence of Sex Hormones on Other Risk Factors for HCC

In addition to acting directly on the risk of developing HCC, sex hormones are also able to modulate the action of other factors and cofactors of liver damage, significantly influencing their carcinogenic potential.

#### 5.3.1. Chronic Viral Hepatitis

Beyond the epidemiological differences in gender distribution, some evidence suggests that both chronic HCV and HBV infections are associated with a greater probability of developing HCC in males rather than in females. The gender difference in the risk of developing HCC during HBV infection appears to be extremely relevant. Indeed, the male-to-female ratio for HBV-related HCC is significantly higher than that for HCV-related HCC [131]. Overall, the incidence of HCC is 5- to 7-fold higher in male HBV carriers than in female ones, making male gender an important risk factor for HBV-related hepatocarcinogenesis. Since higher serum viral loads are associated with increased risk of HCC, the gender effect could be mediated by higher replicative levels observed in men compared to women [128]. Gender differences in HBV viral load levels could be influenced directly by the regulation of viral gene expression and indirectly by host immune responses modulation. Indeed, estrogens and androgens determine an opposite regulation of HBV transcription [128]. Stimulation of the AR by androgens is able to increase overall HBV transcription [132], and vice versa, hepatitis B virus X protein (HBx), involved in viral replication mechanisms, has been shown to increase hepatic AR activity in an androgen-dependent manner [133]. Indeed, a positive cycle is created that is able to aberrantly activate and maintain the activity of hepatic RA, elevate viral replication levels, and enhance the oncogenic risk in male patients with HBV infection [128]. On the other hand, in light of the evidence that the incidence of HBV-related HCC is more frequent in postmenopausal than in premenopausal women, it has been hypothesized that estrogens may also play a central role in regulating oncogenic risk during chronic HBV infection [134]. In contrast to the viral replication-promoting role of the androgen/AR axis, the action of estrogens results in a reduction in viraemia levels in the host. In particular, estrogens support the hepatic expression of its nuclear receptor ERα, which can suppress the modulating activity of viral enhancer I and consequently reduce HBV transcription [135]. Overall, HBV is therefore considered a sex hormone responsive virus, whose replication is stimulated by androgens and inhibited by estrogens. The different levels of viraemia (higher in humans) resulting from this hormonal effect could explain at least in part the gender differences in the risk of developing HCC during HBV infection. However, in addition to the direct action of sex hormones on the virus life cycle, the modulation of oncogenic risk exerted by sex hormones in HBV-related HCC could also be mediated by the ability to influence the immune response to HBV infection [128]. Thanks to a more intense immune response, clearance of the hepatitis B envelope (HBeAg) and surface (HBsAg) antigen and the relative seroconversion are more frequent in women and the protective response conferred by vaccination is more significant. Although the mechanisms of this disparity are still unclear, it is known that the androgen/AR axis is capable of exerting immunosuppressive effects on the development and activation of T cells [136], amplifying the action already exerted by the virus itself [49]. The higher levels of viral replication secondary to such immunosuppressive effects could be a crucial cofactor for higher risk of HCC in HBV-infected men compared to women.

Similar to what occurs during HBV infection, in chronic hepatitis C, male gender has been shown to be an independent risk factor for faster progression rate towards cirrhosis and consequently for HCC [137]. Similar to HBx for HBV infection, HCV core protein has been shown to increase AR-mediated transcriptional activity via activation of the JAK/STAT pathway [138]. Since the vascular endothelial growth factor (VEGF) is a target gene of AR in the liver and plays an important role in tumor angiogenesis, the increased transcriptional activity of AR leads to higher risk of developing HCC in HCV-infected patients. As concerns female sex hormones, estrogen affects HCV replication through viral interactions with estrogen receptors. In particular, estradiol has been shown to stimulate the production of interferon-γ (IFN-γ), which can inhibit tumor growth [139]. In HCC, IFN-γ indeed induces autophagy processes, determining growth inhibition and cell death through interferon-regulatory factor-1 (IRF-1). Furthermore, the activation of the membrane estrogen receptor GPER is able to increase metallopeptidase MMP-9 levels [140]. The latter has the ability to cleave and block the activity of occludins, structural proteins of membrane tight junctions, used by HCV to enter cells. Therefore, high estrogenic activity, as occurs in premenopausal women, limits the cytolytic and replicative activity of HCV, reduces the rate of liver damage progression, and, at the same time, the risk of HCC.

#### 5.3.2. Obesity

Obesity is an independent risk factor for malignancies, including HCC. As already discussed, to date it is the cause of about 9% of HCC cases worldwide [3], increasing the oncogenic risk by 2 to 4 times in presence of damaging cofactors [39,40]. In addition to the pro-oncogenic risk resulting from the development of nonalcoholic steatohepatitis (NASH) and metabolic cirrhosis, obesity may favor HCC occurrence even in absence of significant liver fibrosis by promoting systemic and hepatic inflammation, inducing oxidative stress and lipotoxicity, stimulating the insulin-like growth factor-1 (IGF-1) axis by hyperinsulinemia, and favoring hormonal changes [141]. In this regard, obesity is associated with high levels of leptin, a hormone produced by adipose tissue and the small intestine, which is crucial in regulating mechanisms of energy balance and body weight control [142]. Although, at the central level, it influences the hypothalamic-pituitary-adrenal axis by regulating feelings of hunger, in the periphery, it is able to influence the reproductive system, the basal metabolic rate, the production and sensitivity to insulin, and regulate both the innate and acquired immunity. As a compensatory mechanism to preserve insulin sensitivity, leptin levels increase with increasing fatty mass, but persistent hyperleptinemia is associated with more severe liver steatosis and is involved in fibrinogenesis and hepatocarcinogenesis processes [143,144]. In addition to its role as regulator of energy balance, leptin can in fact act as a growth factor and promote the development of neoplasms. In the liver, it has been shown to promote the development of HCC, as well as its progression, invasiveness, and migration through the activation of the JAK/STAT pathway [145]. In light of the higher incidence of HCC in men than in women, a potential inhibitory effect of estrogens on leptin-induced HCC has been hypothesized. Shen et al. [146] demonstrated that 17β-estradiol is able to suppress leptin-induced liver tumor cell proliferation and promote cell apoptosis. This effect is achieved through estrogen binding to both ER-β, with consequent reversal of leptin-induced changes in SOCS3/STAT3 and p38/MAPK activation, and ER-α, as well as to GPER, with secondary activation of the ERK pathway. The overall effect of estrogen would therefore be to antagonize the oncogenic actions of leptin.

Similar to leptin, adiponectin is another hormone produced by adipose tissue and its role is crucial in the mechanisms of metabolism regulation, sensitivity to insulin action, and inflammation [147]. In the liver, adiponectin has demonstrated an antisteatotic action, stimulating the beta-oxidation of fatty acids and reducing tumor necrosis factor (TNF)-α circulating levels [148]. Furthermore, it is protective against HCC development through the activation of AMP-activated protein kinase (AMPK) [149]. Serum adiponectin levels tend to decrease in the case of insulin resistance, such as in obesity and/or diabetes mellitus [147], and in males [150]. Compared to women, serum adiponectin levels are significantly reduced in men. In conditions of obesity and insulin resistance, the reduction in adiponectin seems responsible for the increased HCC risk in males [149]. Since higher adiponectin levels have been found after castration in males, it seems likely that androgens are the most responsible for gender disparities in adipokine concentrations. In particular, testosterone could activate the c-Jun N-terminal kinases (JNK) protein, resulting in inhibition of adiponectin secretion and increased risk of HCC.

### 5.4. Interaction between Sex Hormones and miRNAs

Gender differences in the expression of microRNAs have recently emerged (miRNAs) in the context of HCC and in the influence exerted on them by sexual hormones. MiRNAs are small, noncoding, single-stranded RNAs that play the role of posttranscriptional regulators of protein encoding genes [151]. They interact with the 3′ region of the target mRNA influencing its transcription processes. Changes in miRNA expression are crucial in the regulation of complex genetic networks and cellular signaling cascades. At the same time, altered miRNA expression plays a central role in the regulation of protein expression within the pathological changes of numerous diseases. For example, several miRNAs (e.g., miR-122, miR-21, miR-34a, miR-451) are enhanced in patients with NAFLD [152]. Aberrant miRNA expression can be frequently encountered in several human cancers. Genomic regions encoding miRNAs can protect against genetic mutations, whereas carcinogenesis-related transcription factors can suppress some miRNAs and favor the development of pro-oncogenic mutations [153]. In recent years, numerous data have emerged on the role of miRNAs in the genesis and progression of liver cirrhosis and in HCC occurrence [154]. In the liver cancer setting, several HCC-associated miRNAs (miR-21, miR-221, miR-222) are increased, whereas others (miR-122a, miR-145, miR-199a, miR-223) are decreased. Indeed, healthy hepatocytes and HCC cells express different miRNA profiles [155].

Recently, significant gender differences in miRNA expression have emerged in patients with HCC, with close correlation with sex hormones. Among others, miR-216a appears significantly upregulated in HCC cells, particularly in male patients [156]. Through AR, androgens are able to bind AREs in the promoter region of pri-miR-216a and determine a significant increase in its transcription. Moreover, during chronic HBV infection, the HBx viral protein is able to further enhance the AR-mediated protranscriptional effect. Unlike the male prevalence of mir-216a, miR-18a appears significantly increased in women with HCC (female/male ratio: 4.58) [157]. miR-18a is able to bind to the 3′UTR region of the ESR1 gene mRNA which codes for the ERα estrogen receptor, inhibiting its transcription. In HCC cells, overexpression of miR-18a decreased ERα levels. Therefore, it inhibits the protective effects of estrogen, promoting hepatocarcinogenesis in women.

A similar action is also performed by miR-22 [158]. This miRNA is in fact able to inhibit the activity of ERα through binding to the 3′UTR region of its mRNA, compromising the estrogen signaling cascade. In male HBV-infected patients, miR-22 has been shown to promote the development of HCC [159]. Indeed, overexpression of miR-22 in male HBV-related HCC adjacent tissue correlates with downregulated ERα. This phenomenon could mitigate the protective effect of estrogens on HCC occurrence in male HBV-infected patients. The downregulation of ERα secondary to miR-22 overexpression could also lead to an increase in IL-1α expression. The latter is a cytokine released in response to hepatic necrosis able to determine a compensatory proliferative response [160]. Its increase could further contribute to loss of estrogen’s protective mechanisms against the development of HCC [159].

Zhao et al. [161] finally evaluated the expression of the miR-545/374a cluster in the HBV-related HCC setting. In fact, in the presence of the viral protein HBx, there is a significant increase in miR-545/374a expression in males (but not in females) with HBV-related HCC. Encoded by the Ftx gene, these miRNAs are overexpressed in HCC secondary to HBV infection and are associated with poorer prognosis. Furthermore, estrogen-related receptor gamma (ESRRG), a protein belonging to the ER-like receptor family, is inversely correlated with miR-545 expression. However, its role in the development of HCC has not been clarified yet.

## 6. Therapeutic Implications

Since sex hormones play a central role in the development of HCC, numerous attempts have been carried out to identify an estrogenic or anti-androgenic therapy able to improve its prognosis. In the 1990s, on the back of other therapies for estrogen-dependent tumors, many studies focused on the use of tamoxifen, a selective estrogen receptor inhibitor (SERM), in the HCC setting. Hypothesizing that estrogens promote hepatocyte proliferation and hepatocarcinogenesis, Martínez Cerezo [162] and Farinati [163] et al. experimented with the use of tamoxifen in small groups of patients with liver cirrhosis and unresectable HCC. In both studies, anti-estrogen therapy with tamoxifen appeared to significantly prolong survival compared to untreated controls. Subsequent randomized placebo-controlled trials instead overturned the hypothesis of the efficacy of tamoxifen in patients with HCC. They found that tamoxifen administered in 329 patients with unresectable HCC, at doses of 60 mg/day and 120 mg/day, was associated with an increase in mortality compared to placebo [164]. The reduction in survival was proportional to increasing dose, underlining its negative impact on the prognosis of HCC patients. Subsequently, the CLIP-1 trial confirmed the ineffectiveness of tamoxifen in a subgroup of patients with early-stage HCC [165]. According to Wu et al. [166], the sensitivity of HCC to the action of tamoxifen could be related to the nuclear expression of ERα in hepatocytes. In patients with high nuclear expression of this receptor, tamoxifen therapy could be effective. To date, however, there are no data to support its use. Similarly to tamoxifen, megestrol, a synthetic derivative of progesterone, has also demonstrated no clear benefits in patients with unresectable HCC [167].

Numerous studies have focused on the potential role played by menopausal hormone therapy (MHT) in the occurrence of HCC in women. McGlynn et al. [168] showed in a case-control study that MHT is significantly associated with lower risk of HCC, particularly among women receiving estrogen only MHT. Similarly, Hassan et al. [24] confirmed the protective role of estrogen treatment on the development of HCC in women, estimating a reduction in the risk of liver cancer equal to about 50%. Moreover, in women with HCC, MHT appears to be associated with a significant increase in OS compared with controls (33.5 months for estrogen users and 24.1 months for nonusers). However, the benefits obtained in terms of HCC from a MHT that includes only estrogen use is counterbalanced by a significant increase in the risk of breast, ovarian, and endometrial cancer [169], which makes its use in prophylaxis unacceptable. However, its potential role in the treatment of HCC remains to be clarified.

In addition to estrogenic therapies, the use of anti-androgen therapies has also been evaluated in the treatment of HCC. In this regard, the use of the anti-androgens flutamide [170] and cyproterone acetate [171], of ketoconazole [172], and of D-tryptophan-6-luteinizing hormone-releasing hormone (analogue of luteinizing hormone-releasing hormone capable of inhibiting the pituitary-gonadal axis) [173] has given extremely disappointing results. Despite the historical ineffectiveness of these approaches, recently, it has been hypothesized that the absence of benefits provided by anti-androgen therapy may be related to spliced variants of the androgen receptor, called AR-SV [174]. Indeed, AR-SV expression in liver cancer could favor HCC progression by regulating the epithelial-to-mesenchymal transition pathway and determining resistance to traditional AR antagonists. A selection of patients who do not express such variants could allow researchers to determine the potential benefits from anti-androgen therapy in the HCC treatment setting [175]. A potentially effective strategy to overcome this drug resistance could be the selective blockade of AR-SV. Selective androgen receptor degraders (SARDs) could be used in this regard, both in the setting of prostate cancer [176] and HCC. Furthermore, recent evidence identifies mechanisms of feedback activation of the AKT-mTORC1 pathway (a major oncogenic pathway central to the processes of hCC development) in the setting of enzalutamide-treated HCCs [126]. Therefore, treatment with AR blocking drugs activate compensatory mechanisms that mediate intrinsic resistance to AR antagonists, providing a potential further explanation for the lack of efficacy of anti-androgen monotherapy. Combination therapies able to simultaneously block both the AR and AKT-mTORC1 pathways should be evaluated in the treatment of HCC [175].

Despite the significant epidemiological and prognostic differences, clinical and therapeutic applications in the HCC setting are still scarce. In fact, although preclinical research has shown that several mechanisms are involved in gender differences, gender-specific therapies are not yet available. It seems promising that everything known about gender differences in HCC could be applied in clinical setting. In fact, compared to other sectors, gender medicine is still underdeveloped today. In the HCC setting, significant gender differences and underlying mechanisms could be used to improve anti-cancer therapies available today. In fact, because of the low efficacy of current treatments for HCC and the central role played by sex hormones in hepatocarcinogenesis, hormonal therapies could be part of combination therapy schemes (systemic and/or loco-regional) potentially able to improve objective treatment response and OS and reduce the risk of recurrence. Specific clinical trials are missing and therefore mandatory.

## 7. Conclusions

The liver is a sexually dimorphic organ with large differences in gene expression, cellular function and composition, and immune response. Large disparities are consequently found in the organ’s responses to pathogenic noxae. In women, estrogens play a protective role, limiting liver inflammation and fibrogenesis and counteracting the development of HCC. They are also partially responsible for the lesser aggressiveness of liver cancer, as well as better response to treatments, lower recurrence rates, and an overall better prognosis. Conversely, androgens promote cell proliferation and hepatocarcinogenesis, elevating the risk of developing HCC. In addition to direct action, sex hormones influence oncogenic risk by modulating other risk factors’ activity (e.g., HBV infection, obesity, and metabolic syndrome). Hence the need for even closer and more effective surveillance in the male gender, which is at greater risk of occurrence and recurrence of HCC. However, more research on gender is needed to investigate mechanisms underlying differences in the pathogenesis of HCC. To date the use and/or manipulation of sex hormones has not demonstrated efficacy in the treatment of HCC despite their central role. A more in-depth knowledge of the mechanisms linking sex hormones to hepatocarcinogenesis could also mean significant progress in the treatment of liver cancer.

## Figures and Tables

**Table 1:** Risk factors, clinical features, and treatment response of HCC: gender differences.

		Men	Women	Comments
Prevalence of risk factors	HCV	Lower	Higher	Wide regional variations
	HBV	Higher	Lower	Wide regional variations
	Alcohol	Higher	Lower	Women show more susceptibility to alcohol-related liver injury than men
	Obesity	Lower	Higher	The gender gap is greatest in low-income countries. Visceral obesity is significantly more frequent in men than in women.
	T2DM	Higher	Lower	
	PBC/AIH	Lower	Higher	The overall incidence rate of PBC/AIH is low.
	Smoke	Higher	Lower	The gender gap is greatest in Asia and Africa.
Clinical features	Occurrence	Higher	Lower	Occurrence up to 5 times higher in men, depending on region and clinical
	Age of development	Earlier	Later	Peak incidence in men aged 50 to 69 years. In women similar incidence between 50–69 years and > 69 years.
	Size (diagnosis)	Larger	Smaller	Male gender is independently associated with a more advanced stage of HCC at diagnosis (size, vascular invasion, multifocality, metastasis).
	Encapsulation rate (diagnosis)	Lower	Higher	
	Multifocality rate (diagnosis)	Higher	Lower	
	Vascular invasion (diagnosis)	Higher	Lower	
	Metastasis rate (diagnosis)	Higher	Lower	
	Overall stage (diagnosis)	Most advanced	Earliest	
	Course	More aggressive	Less aggressive	
	Prognosis	Worst	Better	The mortality rate is 2–3 times higher in men than in women. Female gender is independently associated with lower mortality rate.
Treatment response	Response to treatment	Worst	Better	Female gender is independently associated with a higher first therapy response rate.
	Recurrence rate	Higher	Lower	Male gender is an independent risk factor for early recurrence (OR: 1.864). DFS is, on average, higher in women than in men (4.5 vs. 19.5 months).

AIH: autoimmune hepatitis; DFS: disease free survival; HBV: hepatitis B virus; HCV: hepatitis C virus; PBC: primary biliary cholangitis; T2DM: type 2 diabetes mellitus.

## Data Availability

Not applicable.
